# Neutralizing and Total/IgG Spike Antibody Responses Following Homologous CoronaVac vs. BNT162b2 Vaccination Up to 90 Days Post-Booster

**DOI:** 10.3390/antib11040070

**Published:** 2022-11-08

**Authors:** Chin Shern Lau, John Thundyil, May Lin Helen Oh, Soon Kieng Phua, Ya Li Liang, Yanfeng Li, Jianxin Huo, Yuhan Huang, Biyan Zhang, Shengli Xu, Tar Choon Aw

**Affiliations:** 1 Department of Laboratory Medicine, Changi General Hospital, Singapore 529889, Singapore; 2 Medical Affairs, Abbott, Singapore 189352, Singapore; 3 Department of Infectious Diseases, Changi General Hospital, Singapore 529889, Singapore; 4 GenScript Biotech (Singapore) Pte Ltd., Singapore 349248, Singapore; 5 Singapore Immunology Network, Agency for Science, Technology and Research, Singapore 138648, Singapore; 6 Department of Physiology, Yong Loo Lin School of Medicine, National University of Singapore, Singapore 117599, Singapore; 7 Department of Medicine, National University of Singapore, Singapore 117599, Singapore; 8 Academic Pathology Program, Duke-NUS Medical School, Singapore 169857, Singapore

**Keywords:** SARS-CoV-2, vaccination, antibodies, BNT162b2, CoronaVac

## Abstract

Introduction: We documented the total spike antibody (S-Ab), IgG S-Ab and neutralizing antibody (N-Ab) responses of BNT162b2/CoronaVac vaccinees up to 90 days post-booster dose. Methods: We included 32 homologous regimen CoronaVac vaccinees and 136 BNT162b2 mRNA vaccinees. We tested their total S-Ab (Roche), IgG (Abbott) and N-Ab (Snibe) levels at set time points from January 2021 to April 2022. All subjects were deemed to be COVID-19-naïve either via clinical history (CoronaVac vaccinees) or nucleocapsid antibody testing (BNT162b2 vaccinees). Results: All antibodies peaked 20–30 days post-inoculation. In BNT162b2 vaccinees, all post-booster antibodies were significantly higher than second-dose peaks. In CoronaVac vaccinees, IgG showed no significant differences between peak third-/second-dose titers (difference of 56.0 BAU/mL, 95% CI of −17.1 to 129, *p* = 0.0894). The post-vaccination titers of all antibodies in BNT162b2 vaccinees were significantly higher than those in CoronaVac vaccinees at all time points. Post-booster, all antibodies declined in 90 days; the final total/IgG/N-Ab titers were 7536 BAU/mL, 1276 BAU/mL and 12.5 μg/mL in BNT162b2 vaccinees and 646 BAU/mL, 62.4 BAU/mL and 0.44 μg/mL in CoronaVac vaccinees. Conclusion: The mRNA vaccine generated more robust total S-Ab, IgG and N-Ab responses after the second and third vaccinations.

## 1. Introduction

Vaccination against SARS-CoV-2 is essential for population control of COVID-19. A mathematical modeling study [1] concluded that vaccinations prevented 14.4 million deaths from COVID-19 across 195 countries from 2020 to 2021, and even today, there is still a need for mass vaccination programs in developing countries. The two most common types of vaccines available are the mRNA vaccine (e.g., BNT162b2 and Pfizer-BioNTech) and the inactivated virus vaccine (e.g., CoronaVac and Sinovac Life Sciences), with most developing countries predominantly utilizing inactivated virus vaccines due to various factors. However, the effectiveness of vaccine types in the prevention of COVID-19 is variable, with most reporting COVID-19 incidence rate ratios that are higher for inactivated virus vaccines; they may differ by as much as 2.37 vs. 0.42 for inactivated virus vs. mRNA vaccinees [2]. In a recent meta-analysis comparing the efficacy of different vaccines, the Moderna mRNA vaccine had the highest vaccine effectiveness against the prevention of infection (98.1%), while the inactivated vaccine (CoronaVac) was only 65.7% effective [3]. Even among heterologous vaccination regimens that use a combination of different vaccines, a third dose of inactivated vaccine vs. mRNA vaccine (after two doses of inactivated virus vaccine) can have a large difference in antibody response, with a single mRNA vaccine booster generating a 6.9-fold difference in anti-receptor-binding domain IgG antibodies after <85 days [4]. IgG antibodies directed against the receptor-binding domain are essential for immune protection, with IgG binding to the spike protein being the neutralizing antibody against the virus and having the greatest correlation with total immunoglobulin binding and neutralization activity [5], presumably by blocking receptor-binding domain sites from interacting with ACE2 to enter the cell [6]. It is, thus, essential to know the longer-term trends of different antibody responses to different vaccines over time. In our previous study [7], we demonstrated that there was a significant difference in the early total spike antibody (S-Ab) and neutralizing antibody (N-Ab) responses between BNT162b2 and CoronaVac vaccinees up to 20 days after the booster dose. However, there is a paucity of data of the extended antibody kinetics more than 60 days after the third vaccination between the two vaccine types. Thus, we report on the antibody levels of healthcare workers up to three months after their third vaccination and compare the difference in patterns between BNT162b2 and CoronaVac vaccinees.

## 2. Methods

### 2.1. Study Participants

We included 32 subjects who received 3 doses of CoronaVac inactivated virus vaccine (mean age of 46.1 ± 13.6 years) and 136 subjects who received 3 doses of BNT162b2 mRNA vaccine (mean age of 43.8 ± 13.5 years) and tested their antibody levels from January 2021 to April 2022. All samples were from healthcare workers who were reportedly healthy, and we had immunocompromised subjects. During this period, Singapore experienced two waves of SARS-CoV-2 variants: Delta (August to November 2021) and Omicron (from December 2021 onwards) [8]. BNT162b2 vaccinees were COVID-19-naïve throughout the study, as evidenced by negative total SARS-CoV-2 nucleocapsid antibodies (Roche total anti-SARS-CoV-2 nucleocapsid antibody assay) at all time points. As nucleocapsid antibodies also increase after inoculation with inactivated virus vaccines [9], CoronaVac vaccinees were deemed COVID-19-naïve based on the absence of symptomatic disease throughout the study. Antibodies were tested at set time points up to 90 days post-booster vaccination. The numbers of samples differed at each time point due to variations in vaccination schedules among participants. All samples obtained in this study were de-identified and anonymized.

### 2.2. Methods and Materials

Serum samples were frozen at −70 °C if not immediately analyzed. The Roche Elecsys Anti-SARS-CoV-2 S assay (performed on an Elecsys e801 auto-analyzer), the Abbott quantitative IgG assay (Abbott SARS-CoV-2 IgGII; performed on the Alinity immunoassay platform) and the Snibe quantitative N-Ab assay (performed on a Maglumi 2000 analyzer) were previously described in prior studies from our laboratory [7,10]. Although the Roche total S-Ab assay reports titers in U/mL, this can be converted to WHO international units (BAU/mL = 0.97 × U/mL). Similarly, Abbott IgG S-Ab is reported in AU/mL, which can be converted to WHO units (BAU/mL = 0.142 × AU/mL).

### 2.3. Statistical Analysis

Data were presented as medians, with ranges where appropriate. No indeterminate or missing results were used. Mann–Whitney U testing was used to compare the antibody titers between vaccination groups and among time points, with *p* < 0.05 being considered statistically significant. Statistical analyses were performed using MedCalc Statistical Software (version 20.008; MedCalc Software Ltd., Ostend, Belgium). This work was part of a seroprevalence survey using de-identified, anonymized samples/data; it was, thus, exempt from the approval of our hospital’s Institutional Review Board. However, informed consent was still obtained from all subjects involved, as they needed to provide blood samples at several time points. Compliance with STARD guidelines is enclosed (see Supplementary Table S1).

## 3. Results

**Figure 1:**
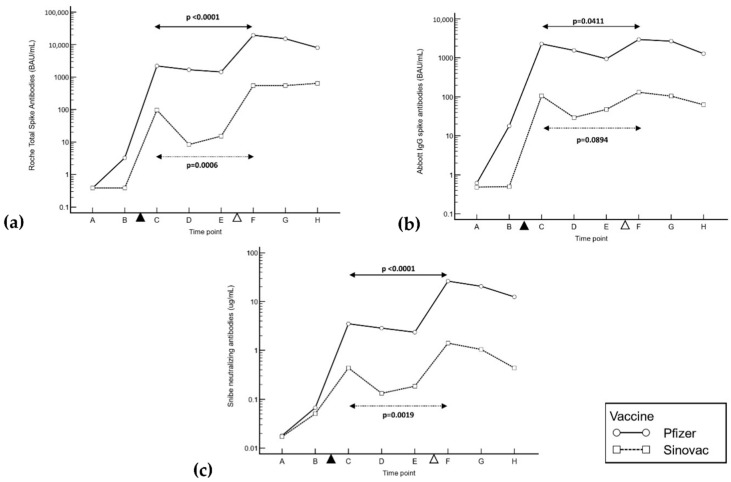
(**a**) Roche total, (**b**) Abbott IgG and (**c**) Snibe neutralizing antibody responses to 3 doses of BNT162b2 vs. CoronaVac inactivated virus vaccines at the same time points. Time points are represented by A, baseline; B, 10 days post-dose 1; C, 20 days post-dose 2; D, 40 days post-dose 2; E, 60 days post-dose 2; F, 20–30 days post-dose 3; G, 50–70 days post-dose 3; H, 90 days post-dose 3. The black triangle represents dose 2, and the white triangle represents dose 3. Antibody levels are expressed on a semi-logarithmic scale. Mann–Whitney U test two-tailed probability between peak antibody levels is displayed for each group.

Population demographics, as well as vaccination intervals and intervals between doses and sampling points, are reported in Table 1. In both groups of vaccinees, all antibodies peaked 20–30 days post-inoculation (see Table 2). All post-third-dose antibody titers were significantly higher than the second-dose peaks in BNT162b2 vaccinees. However, in CoronaVac vaccinees, although the peak total S-Ab and N-Ab responses were significantly higher after dose 3 (total S-Ab difference of 458 BAU/mL, 95% CI of 218 to 1126, *p* = 0.0006; N-Ab difference of 0.83 μg/mL, 95% CI of 0.34 to 1.54, *p* = 0.0019), the IgG responses showed no significant differences between peak titers (difference of 56.0 BAU/mL, 95% of CI −17.1 to 129, *p* = 0.0894) (see Figure 1). Furthermore, apart from N-Ab after the first vaccination, the titers of all antibodies in the BNT162b2 group were significantly higher than those in the inactivated virus vaccinees at all time points (see Table 2). The delay in the rise of N-Ab after the first inoculation has also been noted in other studies [11], with median N-Ab (also Snibe) of only 0.119 μg/mL 12 days after the first dose, only increasing to a peak of 3.39 μg/mL after 28 days. The fold difference in peak antibody levels between the BNT162b2 and CoronaVac vaccinees also increased from the second vaccination to the booster dose: total S-Ab, 23- vs. 35-fold; IgG, 21- vs. 22-fold; N-Ab, 9- vs. 19-fold. Post-booster, all antibodies declined by 90 days in the BNT162b2 group. IgG and N-Ab also declined post-booster in the CoronaVac group; although the median total S-Ab seemed to increase between days 50–70 and 90 post-booster (590 and 646 BAU/mL), this was not statistically significant (difference of 12.1 BAU/mL, *p* = 0.83).

## 4. Discussion

The antibody responses generated by the BNT162b2 vaccines were significantly higher than those in CoronaVac vaccinees at all time points. Indeed, peak IgG antibody levels between the second and third CoronaVac doses showed no significant differences from each other. This may be because of a lesser degree of memory B-cell formation in inactivated virus vaccinees. In one study [12], the measured percentages of B.1 spike-binding and receptor-binding domain-binding memory B cells correlated with N-Ab and S-Ab titers, with the highest percentage of memory B cells being found in BNT162b2 vaccinees (0.022%) and the lowest in CoronaVac vaccinees (0.003%). Other studies also support our findings. In one study of vaccine response in multiple sclerosis patients being treated with disease-modifying agents, mean IgG S-Ab was also demonstrated to be higher in mRNA vaccinees than inactivated virus vaccinees 2 weeks after the second vaccination, regardless of whether they were being treated with disease-modifying agents or not [13]. This pattern was also noted when comparing age groups after 2 doses of vaccine. In patients with an initial two doses of inactivated virus vaccine, boosting with an mRNA vaccine produced an 8-fold rise in IgG, compared with a 3-fold rise obtained with an inactivated virus vaccine [14]. In a Brazilian study [15], 4 weeks after the booster, patients who initially received two doses of CoronaVac had a 152-fold increase in IgG antibodies after an mRNA booster, but only a 12-fold rise after a CoronaVac booster. Furthermore, the homologous CoronaVac vaccination did not elicit 100% seropositivity in older adults (>60 years old). Although it is known that inactivated virus vaccines generate a more robust nucleocapsid antibody response than mRNA vaccines [9], this does not seem to translate into increased real-world effectiveness [3]. Indeed, vaccine effectiveness against COVID-19 was reported to only be 65.9% after two doses of inactivated virus vaccine [16].

All antibodies declined with time after peaking 20–30 days after a new inoculation, even in the CoronaVac group, where IgG/N-Ab declined, and total S-Ab plateaued at a lower level. Other studies [17] also noted that there can be differences in the pattern of decline in virus neutralizing antibodies. The concern of waning antibody levels would be even more pertinent for vaccinees who have received the inactivated virus vaccine, especially when the peak post-booster antibody levels were much lower than mRNA vaccinees. The decline in antibody levels is generally associated with a decline in vaccine effectiveness. For mRNA vaccines, the waning of effectiveness against hospitalization during the Omicron wave was seen 3–4 months after two doses of vaccine (decreasing from a peak of 80.3% to 56.3%) and after the third dose of vaccine (from 81.6% to 50.0%) [18]. In one study [19], after the second dose of inactivated virus vaccine, the odds ratio of having symptomatic COVID-19 increased with time across age groups. Between 0–13 days and >180 days, the odds in 18–39-year-olds were 1.41 to 1.67, but they were 1.22 to 1.65 in those > 80 years old. In a study of the effectiveness of heterologous vs. homologous booster regimens, a homologous inactivated virus vaccine booster only resulted in a vaccine effectiveness of 15.0% against symptomatic COVID-19 8–59 days post-vaccination compared with 56.8% when given an mRNA vaccine booster, with higher vaccine effectiveness against hospitalization and death ≥ 60 days after the booster in heterologous vaccinees (78.5% vs. 51.4%) [20]. In combination, these studies seem to indicate that the declining antibody responses in inactivated virus vaccinees are associated with a decline in vaccine efficacy over time. This may indicate that additional vaccinations may be required, even after an inactivated virus vaccine booster.

Another concern is whether the different vaccines can provide protection against new variants of concern, such as the Omicron variant. One study [21] showed that homologous mRNA vaccine regimens not only produce much higher peak median levels of IgG 4 weeks after the booster but also generate a greater neutralizing antibody against the Omicron variant. In one local study [8] that determined vaccine efficacy during the Omicron wave in Singapore (December 2021), mRNA boosters again showed increased effectiveness in preventing COVID-19 15–60 days after boosting (31.7–41.3%), even when using heterologous mRNA vaccine regimens. On the other hand, a homologous inactivated virus vaccine regimen only had a vaccine effectiveness of 7.26% 15–60 days post-booster.

Thus, we report the following new findings:

- After the second and third inoculations, inactivated virus vaccines produced a much lower post-vaccination antibody response than mRNA vaccines considering all antibodies (total S-Ab, IgG and N-Ab), even 90 days post-booster.
- Aside from IgG in inactivated virus vaccinees, the peak antibody titers increased after booster vaccination in both vaccination groups.
- In all vaccinees, total S-Ab, IgG and N-Ab antibody levels declined up to 90 days post-booster.

A limitation of our study was that we had a small number of subjects who received inactivated virus vaccinations at certain time points. However, the antibody kinetics after vaccination with inactivated virus found in our study is in agreement with the findings of other studies that examined 3 doses of inactivated virus vaccine [22]. We did not have access to virus neutralization assays. In studies of virus neutralization assays, some proposed that a possibly protective virus neutralization titer could be 1:33 [23]. However, in studies that analyzed the neutralization titers [24], a booster dose of 3 ug of inactivated virus vaccine (CoronaVac) elicited a titer of 1:36.4 180 days post-booster, with a 6 ug dose of CoronaVac generating titers of 1:62.8; both titers were lower than the proposed protective level. In adults aged > 60 years old, a 3 ug booster only resulted in a titer of 1:29.6 by day 180, which was lower than the protective threshold [24]. As our study did not use any large clinical data set, we had no reliable way to correlate the antibody titers with vaccine effectiveness. However, there are studies that showed a similar pattern in the decline of IgG S-Ab with vaccine effectiveness, with Spearman’s rank correlation coefficient as high as 0.93 [25]. Although the dosing intervals between the first and second dose in both groups was fairly similar (median of 21.0 days), the interval between the second and third CoronaVac doses was shorter than that in the BNT162b2 group (medians of 93.0 vs. 239.5 days). However, our findings are supported by another study [26] that compared the 50% plaque reduction neutralization antibody titers to the Omicron variant between homologous BNT162b2 and CoronaVac vaccinees 3–5 weeks after boosting. BNT162b2 boosting demonstrated protective levels in 22/25 vaccinees (geometric mean titer of 77.8), but CoronaVac boosting failed to achieve this (1/30 vaccinees, with geometric mean titer of 8.9). This was conducted with slightly different dosing intervals of 61–160 days (CoronaVac) and 180–234 days (BNT162b2) between the second and third doses. In another study where COVID-19-naïve subjects (101 CoronaVac and 114 BNT162b2) received a homologous booster >6 months after their second vaccination (median days between first and second doses/second and third doses: CoronaVac, 27/232; BNT162b2, 21/222) [27], the geometric mean titers of N-Abs also showed responses similar to those in the vaccinees in our study, with 1- vs. 13-fold increases in N-Abs against the Omicron variant in CoronaVac vs. BNT162b2 vaccinees. Thus, even with different dosing intervals, the differences between CoronaVac and BNT162b2 antibody responses seem to follow a similar trend. Our study did not have any access to the clinical data of our subjects, and we were unable to assess the impact of underlying diseases on the antibody kinetics.

## 5. Conclusions

In conclusion, the mRNA vaccine generated more robust total S-Ab, IgG and N-Ab responses after the second and third vaccinations, with higher antibody levels even after the natural decline after 90 days. The low antibody titers 3 months post-vaccination in subjects who have received a homologous inactivated virus vaccine regimen may require further booster doses with homologous/heterologous vaccines.

## Figures and Tables

**Table 1:** Age and gender of study population and time interval between doses and sampling.

Population Characteristic	BNT162b2 Vaccinees	CoronaVac Vaccinees
Mean Age (range, SD)	43.8 (22–90, 13.5)	46.1 (22–68, 13.6)
Percentage ≥ 60 years old	16.18% (22/136)	18.75% (6/32)
Gender	39 male, 97 female	6 male, 11 female, 15 unknown
	**Median days (IQR)**	
Interval between dose 1 and 2	21.0 (21.0 to 21.0)	21.0 (21.0 to 21.8)
Interval between dose 2 and 3	239.5 (231.0 to 254.0)	93.0 (93.0 to 100.5)
Dose 1 to time point B (10 days post-dose 1)	10.0 (10.0 to 12.0)	11.0 (10.5 to 13.0)
Dose 2 to time point C (20 days post-dose 2)	21.0 (20.0 to 22.0)	20.5 (19.5 to 21.5)
Dose 2 to time point D (40 days post-dose 2)	40.0 (40.0 to 43.0)	40.5 (40.0 to 41.0)
Dose 2 to time point E (60 days post-dose 2)	61.0 (60.0 to 62.0)	60.0 (60.0 to 60.0)
Dose 3 to time point F (20–30 days post-dose 3)	26.5 (19.0 to 31.0)	20.0 (20.0 to 22.0)
Dose 3 to time point G (50–70 days post-dose 3)	59.5 (57.0 to 61.0)	70.0 (54.3 to 70.0)
Dose 3 to time point H (90 days post-dose 3)	89.5 (88.0 to 92.0)	90.0 (90.0 to 90.5)

**Table 2:** Differences in median total, IgG and neutralizing antibodies at each time point between both groups of vaccinees.

Time Point	Roche Total Antibodies			Abbott IgG			Snibe Neutralizing Antibodies		
	Pfizer (BAU/mL)	Sinovac (BAU/mL)	Difference (BAU/mL) (*p* Value)	Pfizer (BAU/mL)	Sinovac (BAU/mL)	Difference (BAU/mL) (*p* Value)	Pfizer Median N-Ab (μg/mL)	Sinovac Median N-Ab (μg/mL)	Mann–Whitney U Difference (μg/mL) (95% CI)
10 days post-dose 1	3.29	0.39	2.29 (*p* < 0.0001)	17.8	0.50	17.2 (*p* < 0.0001)	1.71	0.22	0.019 (*p* = 0.1191)
20 days post-dose 2	2219	97.0	2079 (*p* < 0.0001)	2271	106	2177 (*p* < 0.0001)	3.52	0.44	2.87 (*p* < 0.0001)
40 days post-dose 2	1695	13.0	1682 (*p* = 0.0173)	1547	29.5	1509 (*p* = 0.0174)	2.84	0.14	2.68 (*p* = 0.0173)
60 days post-dose 2	1454	94.9	1373 (*p* = 0.0173)	941	48.0	898 (*p* = 0.0173)	2.35	0.21	2.17 (*p* = 0.0173)
20–30 days post-dose 3	19,562	555	18,285 (*p* < 0.0001)	2932	131	2685 (*p* < 0.0001)	26.4	1.41	24.4 (*p* < 0.0001)
50–70 days post-dose 3	14,992	590	14,403 (*p* = 0.0055)	2659	105	2554 (*p* = 0.0055)	20.6	1.05	19.6 (*p* = 0.0055)
90 days post-dose 3	7532	646	7101 (*p* = 0.0001)	1276	62.4	1174 (*p* = 0.0001)	12.5	0.44	11.6 (*p* = 0.0037)

## Data Availability

All related data and methods are presented in this paper. Additional inquiries should be addressed to the corresponding author.

## Supplementary Materials

The following supporting information can be downloaded at: https://www.mdpi.com/article/10.3390/antib11040070/s1, Supplementary Table S1: STARD checklist.

## Abbreviations

S-Ab: spike antibodies. N-Ab: neutralizing antibodies.
