# Supplementary material for: Neutralizing and Total/IgG Spike Antibody Responses Following Homologous CoronaVac vs. BNT162b2 Vaccination Up to 90 Days Post-Booster
Source: Antibodies (Basel). 2022 Nov 8;11(4):70. doi: 10.3390/antib11040070 (PMC9680337; doi:10.3390/antib11040070)
Supplement: Supplementary file 1 [file antibodies-11-00070-s001.zip › antibodies-1980011-supplementary.pdf]

Table S1. STARD checklist.

Section & Topic	No	Item	Reported on page #
**TITLE OR ABSTRACT**			
	**1**	Identification as a study of diagnostic accuracy using at least one measure of accuracy (such as sensitivity, specificity, predictive values, or AUC)	**2**
**ABSTRACT**			
	**2**	Structured summary of study design, methods, results, and conclusions (for specific guidance, see STARD for Abstracts)	**2**
**INTRODUCTION**			
	**3**	Scientific and clinical background, including the intended use and clinical role of the index test	**3**
	**4**	Study objectives and hypotheses	**3**
**METHODS**			
Study design	**5**	Whether data collection was planned before the index test and reference standard were performed (prospective study) or after (retrospective study)	**4**
Participants	**6**	Eligibility criteria	**4**
	**7**	On what basis potentially eligible participants were identified (such as symptoms, results from previous tests, inclusion in registry)	**4**
	**8**	Where and when potentially eligible participants were identified (setting, location and dates)	**4**
	**9**	Whether participants formed a consecutive, random or convenience series	**4**
Test methods	**10a**	Index test, in sufficient detail to allow replication	**4**
	**10b**	Reference standard, in sufficient detail to allow replication	**4**
	**11**	Rationale for choosing the reference standard (if alternatives exist)	**4**
	**12a**	Definition of and rationale for test positivity cut-offs or result categories of the index test, distinguishing pre-specified from exploratory	**4**
	**12b**	Definition of and rationale for test positivity cut-offs or result categories of the reference standard, distinguishing pre-specified from exploratory	**4**
	**13a**	Whether clinical information and reference standard results were available to the performers/readers of the index test	**4**
	**13b**	Whether clinical information and index test results were available to the assessors of the reference standard	**4**
Analysis	**14**	Methods for estimating or comparing measures of diagnostic accuracy	**5**
	**15**	How indeterminate index test or reference standard results were handled	**5**
	**16**	How missing data on the index test and reference standard were handled	**5**
	**17**	Any analyses of variability in diagnostic accuracy, distinguishing pre-specified from exploratory	**5**
	**18**	Intended sample size and how it was determined	**Not applicable**
**RESULTS**			
Participants	**19**	Flow of participants, using a diagram	**Not applicable**
	**20**	Baseline demographic and clinical characteristics of participants	**4**
	**21a**	Distribution of severity of disease in those with the target condition	**Not applicable**
	**21b**	Distribution of alternative diagnoses in those without the target condition	**Not applicable**
	**22**	Time interval and any clinical interventions between index test and reference standard	**Not applicable**
Test results	**23**	Cross tabulation of the index test results (or their distribution) by the results of the reference standard	**5**
	**24**	Estimates of diagnostic accuracy and their precision (such as 95% confidence intervals)	**Not applicable**

	**25**	Any adverse events from performing the index test or the reference standard	**Not applicable**
**DISCUSSION**			
	**26**	Study limitations, including sources of potential bias, statistical uncertainty, and generalisability	**9**
	**27**	Implications for practice, including the intended use and clinical role of the index test	**9**
**OTHER INFORMATION**			
	**28**	Registration number and name of registry	**5**
	**29**	Where the full study protocol can be accessed	**Not applicable**
	**30**	Sources of funding and other support; role of funders	**Not applicable**
